# Supplementary material for: Are carbon emissions trading and green financial instruments synergistic? -Comprehensive quantitative research based on content analysis
Source: PLoS One. 2024 Mar 7;19(3):e0298601. doi: 10.1371/journal.pone.0298601 (PMC10919601; doi:10.1371/journal.pone.0298601)
Supplement: S1 Data — (ZIP) [file pone.0298601.s001.zip › Data/地级市Z_F工作报告.zip!/╝¬┴╓/╦─╞╜╩╨/└┤╫╘╣1⁄2╓┌║┼í╛┬φ┐╦╩2╛▌═°í┐_2014.docx]

各位代表：

　　现在，我代表四平市人民政府，向大会报告政府工作，请予审议，并请市政协各位委员提出意见。

　　一、2013年工作回顾

　　2013年是新一届政府开局之年，经济社会发展稳中有进，稳中向好。在省委、省政府和市委正确领导下，在市人大、市政协监督支持下，市政府与全市人民一道，围绕“一核三带”富民优先战略，攻坚克难，奋力前行。预计地区生产总值完成1210亿元，同比增长9.5%；固定资产投资完成735亿元，同比增长24.5%；地方级财政收入完成60亿元，同比增长6%。全面实现“五个着力、两个明确”的主体任务，各项工作取得新的成绩。

　　——着力转变方式，实现工业经济平衡发展。始终把工业作为四平发展的脊梁，采取项目建设、招商引进、股权出让、技术改造、资金注入、市场营销等方式，做大增量、盘活存量、提高质量。实施项目分类管理，坚持要素优化配置，促进谋划、评估、选商、开工等环节衔接，突出“五个重大”，实行市级领导包保，大力开展“项目落实年”、“项目建设百日会战”、“项目服务周”活动。全市新建续建亿元以上项目260个，10亿元以上项目23个。总投资31亿元的双辽迎新玻璃项目，当年开工、当年建设、当年投产，为打造百亿玻璃产业园区奠定坚实基础。公主岭一汽四环工业园、梨树新希望集团农畜科技产业园、伊通石油扩能提产、烟厂30万大箱易地改造等一批重大项目快速推进。实行重大项目专家论证评估，谋划储备一大批亿元以上项目，达到可研程度86个，项目库发挥重要作用。着力推进金融创新，通过建立产业引导基金、设立“中小企业助保金池”、推出注册商标权质押贷款等，积极破解项目建设资金瓶颈。精心组织“商会协会对接年”和“专业小分队招商年”活动，重点围绕专用车、换热器、食品等5个特色产业链，实行定向性、专业化招商和以商招商、小分队招商，先后赴京冀粤港沪等地开展经贸合作交流洽谈，大连万达、河北迎新、浙江中天等多家战略投资者进驻我市。预计全年省外招商引资到位资金630亿元，同比增长25.7%。重点推进企业嫁接改造，中建材控股红嘴水泥，企业规模效益大幅提升；英力建机与柳工集团嫁接，产品市场份额明显扩大；中航奋进项目资金及技术管理团队均已到位，研发能力得以保障，销售额同比大增；一汽专用车与天津中科合作项目正式投产，新产品供不应求，订单成倍增长；中电建线路器材厂易地改造项目已经启动。吉林东北亚通用航空技术有限公司落户四平经济开发区，全省首架通用飞机参展东博会，并签订30架飞机销售意向性协议。铁东奇瑞工业园、中粮生化10万吨果葡糖浆等项目的实施，推动了支柱产业向高端化转型。我市列入“全国老工业基地调整改造规划”，“国家级先进装备制造业新型工业化示范基地”、“全国质量强市示范城市”、国家热交换产品质量监督检验中心通过评审，换热器高新技术特色产业基地获省政府批准。加快发展开发区、工业集中区，推进土地集约、产业集聚、技术集成。双辽经开区、四平循环示范区通过省级开发区审核。全市共实施技改项目240项，完成技改投资246.7亿元。工业投产新产品达163种，其中5种通过省级鉴定，产值221亿元，增长30.2%，新产品产值率达13.3%。依托国家可持续发展实验区争取科技专项资金4650万元。纵深推进产学研合作，积极开展“院士专家四平行”，巨元瀚洋板式换热器和吉春制药鹿产业开发两个院士工作站相继挂牌。市直创新、项目、人才、金融、商务等五个中心启动运行。大力推进节能减排，全市主要污染物总量减排任务圆满完成。

　　——着力示范引领，现代农业加快发展。始终突出品牌农业、效益农业建设，发展特色规模农业、绿色生态农业，提高农业产业化水平。成功抵御春季低温、多湿等不利气候影响，有效防控二代粘虫，积极推进“高光效”、测土配方施肥信息服务等新型实用技术，新建粮食高产示范区100个，粮食总产达到170亿斤，再创历史新高，实现“十连丰”。新建扩建20个棚室蔬菜园区，15个园区被评为国家级、省级标准园。开工建设牧业小区317个，牧业经济保持平稳较快增长。15家企业获得全省“休闲旅游农业企业星级”称号。“公主岭玉米”地理标志成功注册，实现了我市地理标志商标零的突破。巨润生物等7户企业晋升为省级农业产业化重点龙头企业，主推“公司+合作组织+农户”紧密型利益联结模式。全市农民合作社发展到7634个，家庭农场2015户。农村土地承包经营权流转面积达到农户承包地总面积的25.6%。农民土地经营权收益保证贷款扩面提效，农民贷款难问题有效破解。梨树县与中国农大联合创办的产学研基地已经成为“专家农业”模式。

　　——着力创新服务，三经工作卓有成效。始终坚持探索模式创新、组织引导推进，从总部经济、三经协调、民营经济大发展，到今年提出商域港建设，探索经济发展的蓝海。多措并举推动总部经济发展，实行市级领导包保，四个班子领导带领103个部门以小分队形式走出去，与104个行业协会商会对接洽谈，与深圳百泰珠宝等980个国内名牌产品企业取得联系并形成合作意向，与香港豪德、广州云浮签订建立总部基地合作协议。深圳海王药业、广东东鹏陶瓷东北总部落户四平。市人大、市政协机关倾力投入，效果颇丰。预计全市落户总部企业507户，实现税收8.8亿元,实现销售收入240亿元以上，营销范围涉及东三省近三分之二区域。辽河区共引进宇通重工、中铁九局等121户总部企业，实现总部税收9000万元，完成任务指标的129%。提出以“4+2+8+5+4”总部经济服务体系为主体内容的商域港模式，“东北批发在线”电子商务平台业已搭建，探索新兴服务业发展。大连万达城市综合体开工建设，与中行吉林省分行签订金融战略合作协议，铁西九州第五郡商业街区、维美戴斯酒店等高端业态项目提升了四平服务业档次，专用车、换热器对俄、哈销售平台加快推进。把民营经济作为抢占新一轮竞争发展的突破口，开展“六查六比六做”和“万名干部服务万企”活动，出台突出发展民营经济意见，注入5000万元建立民营经济发展专项资金，鼓励民营企业嫁接重组、技术改造、品牌建设等创新发展。全市私营企业、个体工商业户分别发展到1.2万户、14.7万户，比去年底增加1996户、1.7万户。预计民营经济主营业务收入增长15%，实现税金增长6%。

　　——着力梳理改造，城乡环境明显改观。始终坚持“山水林路网”的梳理与提升，注重生态环境建设，做强核心城区，建设特色城镇，打造美丽乡村。着手编制“四梨同城化”规划，编制实施“两河四岸五湖”等城乡建设规划。着眼提升中心城区品质和承载力，加快城市基础设施建设。建设维修主次街路，改造供热和燃气管网120公里，天然气储气量增加14倍，新增供热能力200万平方米。建设东南生态新城精品工程，紫气大路、旭日立交桥竣工通车、四平综合客运枢纽站投入试运营。西南文化宜居新区基础设施多线施工，县(市)新城建设扎实推进。全市完成造林绿化26.1万亩，首创绿化基金，市区建成区绿化覆盖率提高到35%。市区大树进城41.8万株，三年累计完成70.1万株、社会义务投入3900万元、财政及专项投入1000万元，成活率达到90%以上，进一步改善了城市生态环境。积极开展大气污染防治和区域河流综合治理，全年空气质量良好天数不低于345天，四平、梨树污水处理厂提标升级改造项目相继开工建设。我市列入国家“智慧城市示范市”试点。四梨大街建成通车，长平、长双高速公路开工建设，军民合用机场前期工作进展顺利。探索推进“双向城市化、宅基地换房、商贸市场拉动、中心社区承载、城中村改造”五种新型城镇化建设模式。范家屯镇平洋村整体搬迁、入住农村新型社区模式受到高度肯定。大力推进农村人居环境建设，245个村被命名为国家级“美丽乡村”、国家农村环境连片治理示范村，126个村被命名为“吉林省最有魅力休闲乡村”和省级新农村建设示范村。

　　——着力办好实事，人民生活改善提高。始终坚持推进商、居、食、行、乐、教育、医疗、增收、养老、和谐等10个方面民生重点工作，年初确定的50件民生实事全面完成。编制完成市区《商业网点规划》，确定高中低合理布局，建立4家平价副食品商店。进一步规范教育、医疗、小区物业等收费秩序。稳步改善城乡居民居住条件，超额完成省下达的保障性安居工程建设任务。改造棚户区50万平方米、7150套，新建廉租住房6.3万平方米、1530套，完成“暖房子”工程改造110万平方米、惠及居民1.2万户，完成城区D级危房改造1943户，完成国有垦区和农村危房改造2742户。市区新建改造垃圾中转站10座、增设移动厕所100座。创新物业弃管小区管理模式，改造旧小区210万平方米，成立警务室147个、业主委员会341个。市食品安全举报中心投入使用，41个食品安全快速检测站(室)全面启动，开展食品药品专项整治及“放心药店”评选活动，食品药品抽验合格率持续提高，“民生计量”工程惠及千家万户。解决农村10万人饮水不安全问题。优先发展城市公共交通，打造8条精品线路，更新公交车109辆，新增运营里程21公里，在全省“公交便民”考核验收中名列前茅，得到业界表扬肯定。采取分类治理措施，启动对非法营运三轮车专项整治。成功举办第12届中国国际儿童电影节、全国公路自行车公开赛、赴台湾火花展、第七届社区体育大赛、第四届元宵节灯会和焰火晚会等活动，“全国武术之乡”通过检查验收。为乡村、社区、幼儿园、养老院投入健身器材1703件。送戏下乡、农村公益电影放映、文艺演出等群众文化活动丰富多彩。新增6处全国重点文物保护单位。完成市区20所标准化幼儿园建设，义务教育均衡发展，普通高中名校建设实践探索，职业教育资源整合积极推进，“四师一满意”主题教育活动不断深化。多方筹措资金，继续推进全民免费健康体检大行动，完成324万人健康体检，居民电子健康档案建档率达到76%，开创全国地市级城市免费体检先河，为四平人民谋取了健康之举。稳步推进生育关怀、幸福家庭创建、流动人口服务均等化等活动，计生家庭幸福指数普遍提高。深入推进“工资倍增计划”，连续4年提高最低工资标准，连续9年增加退休职工养老金，预计城镇居民人均可支配收入增长11%以上，农民人均纯收入有望突破万元。积极做好创业就业工作，预计全年城镇新增就业6.3万人，城镇登记失业率控制在3.7%。健全完善社会保障体系，社会保险参保人数和覆盖率全面完成省下达任务指标。筹措资金1.47亿元，一次性解决国企改制社保并轨试点失业保险历史遗留问题。建设农村居家养老服务大院84个。76个社区全部建成居家养老服务站，10个社区建成老年人日间照料示范中心，各类养老机构发展到49家。累计向3.23万名残疾人开展了各类康复服务。开展殡葬行业专项整治，减轻群众丧葬负担，树立文明节俭办丧事的新风尚。

　　——明确重点责任，社会局面和谐稳定。始终把人民群众生命财产安全放在首位，面对今年全省安全生产的沉痛教训，坚持安全发展不动摇，组织大量人力物力，大规模强化源头治理，依法严管。在全市范围开展“网络化、无缝隙”安全生产隐患排查治理，广泛开展安全生产标准化企业创建工作，做到坚持标准、突出重点、全域覆盖、明确连锁目标责任、落实技术信息措施、实施依法综合治理，探索建立预警监控平台、责任落实、安全保障、专家排查、协调配合、群众举报、责任追究等长效运行管理机制，着力消除交通运输、易燃易爆、消防以及人群聚集场所等重点领域安全隐患。全市共成立督查检查组289个，督查检查2188次，排查一般隐患43953项、整改37089项；排查重大隐患18项，整改17项，还有1项正在整改中。到目前，全市未发生较大以上安全生产事故，安全生产总体形势良好稳定。稳步推进“法治四平”建设，依法治市工作机制得到完善。深入开展社会管理创新“331”工程，推进“安居乐业美好家园”十星级社区、村创建活动，荣获“全国社会管理创新十佳案例奖”，被授予国家级示范基地。加大矛盾纠纷排查化解力度，编织人民调解防护网，从源头上预防和减少各种矛盾发生。落实信访包案和责任追究制度，一批信访积案得到解决，个体访结案率90%以上，集体访结案率80%以上，交办案件按期结案率100%，切实解决群众信访诉求难题，信访形势好于往年。深入开展平安四平建设，保持严打势头不减，重点打击严重暴力犯罪和多发性侵财犯罪，全面加强治安防控体系“六张网”建设，群众安全感满意度得到提升。

　　——明确职责公开，政府服务提质提效。始终坚持“四型”政府建设，在狠抓落实上下功夫。坚持定期向市委、人大、政协报告通报制度，自觉接受市委领导和人大、政协监督。全年办理人大代表建议97件、政协委员提案224件，办复率达100%。结合政府换届，对责任政府、法治政府、服务政府、廉洁政府建设做出整体安排部署，提高政府执行能力。制定并认真执行新一届市政府工作规则，坚持重大事项集体决策制度，严格按法定程序办事。年初成立6个重点任务推进组，主管市长、主管秘书长对重点任务进行分解，并制成揭示牌送交主管部门落实，共专题调度、调研督查、现场办公127次，解决各类问题159个。依法行政工作深入推进，首次大规模开展市区机关工作人员、行政执法人员和专业技术人员专题培训，行政执法和服务水平不断提高。深入清理行政审批项目，承接省下放项目50项，减少38项，占原项目的14.4%。市政务中心审批效率全面提升，即办件率达到88.9%，累计办理并联审批86件、直通车项目125件。严格落实中央八项规定和省委、市委具体规定，切实克服和解决“五风一顽症”，大力弘扬八种作风，压缩行政支出，对违规人员严肃处理。严格落实党风廉政建设责任制，完善权力运行监控体系，落实一把手监督 “5+1” 制度，加大违法违纪案件查办力度。启动“问政于民、职责公开”工作，利用“两台三报”、政府网站、四平贴吧等10个平台，试行局长“访谈”、副局长“话说”重点工作、科(处)长“答复”群众诉求、一般干部履职评价等方式，畅通与群众联系沟通渠道，及时解决热点难点问题。民族宗教、档案、地震、老龄、地方志、外事侨务、知识产权等工作取得新进展。

　　回顾取得的可喜成绩，主要得益于市委的坚强领导，人大、政协及社会各界的监督支持，得益于政府系统围绕“五个着力、两个明确”付出的不懈努力，得益于企业家、个体工商业者挖掘经营潜能、做大做强企业的创新发展，得益于全市各个方面的智慧汗水和辛勤付出。在此，我代表四平市人民政府，向奋战在全市各条战线上的广大劳动者，向给予政府工作大力支持的人大代表、政协委员、各民主党派、工商联、人民团体和社会各界人士，向关心、支持、帮助四平建设的驻平部队、武警官兵及海内外朋友表示崇高敬意和衷心感谢!

　　在肯定成绩的同时，我们对存在的问题与不足要有清醒的认识。由于宏观经济下行压力增大，部分行业生产经营受到较大影响，个别指标几经努力没能达到年初预计目标，其主要原因是经济发展处于换档期。同时，发展后劲仍然不足，内生增长动力不强；民生投入还不够，一些群众的利益诉求还没有得到很好的解决；发展环境需提高，征地拆迁、劳资关系等影响社会和谐稳定的因素还一定程度存在；部分干部改革创新能力不强、破解难题办法不多、抓落实力度不大等等。对此，一定要引起高度重视，切实采取有效措施，认真加以解决。

　　二、2014年工作安排

　　2014年是全面深化改革的开局之年，也是推进“五城联创”大有作为的一年。2014年政府工作的指导思想是：认真贯彻党的十八届三中全会、省委十届三次全会和市委六届四次全会精神，按照“五个发展”要求，纵深实施“一核三带”富民优先战略，突出“五并重、一加强”，努力打造四平经济升级版，加快在哈大经济大动脉上赶超隆起，全面振兴。

　　坚持稳中求进、改革创新的基本原则，实施“一启动、双推进、三提升”的目标任务。2014年主要预期目标是：全市地区生产总值增长10%；固定资产投资增长14%；全口径、地方级财政收入分别增长5%、8%；社会消费品零售总额增长10%；城镇居民人均可支配收入和农民人均纯收入与经济增长同步；居民消费价格总水平控制在3.5%左右；城镇登记失业率控制在4.5%以内。重点做好以下工作：

　　(一)启动深化改革工作部署，全面推进具体改革措施。紧密结合四平实际，抓住机遇、赢得主动，统筹规划、协同推进，重点突破、先行先试，扎实推进13个方面改革、争取12个探索试点，以“敢闯”的锐气和“会闯”的睿智，实现“百姓富、生态美”有机统一，为全面建成小康社会提供强大动力和体制机制保障。进一步深化农业农村经营体制改革创新，制定农业规模经营的政策意见，积极争取农村集体资产产权改革试点、重点小城镇扩权试点、粮食主产区利益补偿机制和城乡统一的建设用地市场试点、农村宅基地流转试点。进一步深化金融体制机制改革创新，积极推广农村土地收益保证贷款融资模式，争取林场、草场、水库等领域物权融资试点、“粮食银行”试点。深化水、天然气等领域价格改革，完善市场价格形成机制。制定鼓励社会力量兴办教育意见、促进新形势下社会组织健康发展意见、政府向社会力量购买服务的具体实施办法。争取城管体制创新试点、安全监管防控体系建设试点、公共文化服务体系改革试点、基层医疗卫生机构综合改革试点。制定加快技术创新、促进科技成果产业化的意见，争取地方与科研单位建立产学研协同创新机制试点。积极争取突出发展民营经济综合配套改革示范区试点，探索民营经济在市场准入、工商登记、市场监管、金融创新等方面的配套改革措施。制定多渠道增加农民财产性收入的意见，探索建立农村产权流转交易市场。各地各部门要把全面深化改革摆上更加突出位置，结合本地本部门实际，精心谋划、认真研究，积极争取、大胆探索，确保改革举措不折不扣落到实处。

　　(二)推进产业转型升级，提高运行质量和效益。切实优化投资方向，深度谋划一批，集中落地一批，加快建设一批，力促达效一批，加快建设引领产业方向、拉动税源增长、支撑经济发展的重大项目。继续把招商引资作为经济工作的主旋律，推进小分队招商、以商招商、专业化招商，突出产业链条招商，提升产业科技水平和规模能级。重点打造“五个强市”，加快构建具有四平特色的现代产业新格局。扩模提质四平生产。立足粮食、矿产、能源等资源优势，做大淀粉、酒精、糖、啤酒等产品规模，打造四平特色粮食品牌，抓好粮食安全保障能力建设。重点发展新型铝材、塑钢型材、混凝土砌块、镀膜玻璃等建筑材料，大力发展光伏发电、风力发电、石油天然气以及蓝宝石基板等能源产业及电子产品，高标准开发梅花鹿及畜禽加工等系列产品，加快推进四平昊华易地改造、精细化工嫁接改造，打造吉林省特色医药工业园和中药产业研发生产基地。拓展创新四平制造。围绕“产业、集群、园区、企业、项目”五位一体，以换热器、专用车、汽车零部件、农机装备、风机建机、禽类加工设备等优势产品为重点，加快改造升级，增强产业核心竞争力。研究规划通用飞机、旅游房车、组装住宅等产品及项目，向高端、集成、民用方向发展。依托国家可持续发展实验区，大力实施民生科技示范工程，加快建设高新技术产业园区和吉林大学四平科技园，推进生产力促进中心晋升为国家级技术转移示范机构，谋划建立院士、博士、硕士工作站及研究生培养创新实践基地，设立科技创新基金以及公共实验室，提升企业创造力。加快推进开发区、工业集中区转型升级步伐，每个开发区集中力量培育1-2个“竞争力强、成长性好、关联度高”的特色主导产业，推动集群发展、集约发展。构建新兴四平商域港。探索与各级各类行业协会商会合作新路径，下力量引进名牌产品企业东北区域总部，建立总部企业结算中心，提供仓储、分拨、分装、分销条件，推动东北区域总部展销城、总部会馆、东北物流园区建设。探索现代流通方式,加快建设“东北批发在线”电子商务平台，开展网上交易。全力推进万达城市综合体、九洲第五郡商业街、东北珠宝展销城建设，积极筹建五洲国际工业博览城、建材批发市场、特种车交易市场。抓好吉林伊通商贸物流城项目开发。加快推进内外贸一体化发展，支持企业“走出去”建立分销中心、展示中心等营销服务网络。加快推进投融资平台建设，充分发挥市、县(区)平台企业作用，通过发行债券、股权投资，开辟更多直接融资渠道。

　　(三)推进城镇化有序展开，实现城乡一体化特色发展。抓住国家加快新型城镇化建设机遇，因势利导，以人为本，构建特色鲜明、布局合理、功能完善的现代城镇体系。做精做优中心城市。以城市发展总体规划为引领，加快产业布局结构调整，提升“一核”经济实力。加快重点区域改造，抓住国家对棚户区、保障房建设支持政策，加大棚户区改造力度。提升城区主要街路、楼体绿化美化亮化水平，突出小街小巷、城乡结合部治理，抓好以弃管小区为重点的环境整治，让老城旧貌换新颜。高标准建设东南生态新城，建设学校、医院等基础配套工程和“八馆”等公益项目。加快推进“四梨同城化”。抓紧制定发展规划，立即推进工商执照同城通办、电信同号、户籍管理同城、老人同城免费乘车4项工作。以红嘴开发区、四平循环示范区、梨树开发区为依托，大力发展装备制造、循环经济和农畜深加工产业，将市区、梨树县梨树镇、十家堡镇建成交通共联、市场共构、产业共兴的黄金三角区。同时，积极推动伊通与长春融合发展，努力把伊通打造成长春市最美卫星城。做强做特新型城镇。整体设计城镇发展规划，促进城镇空间紧凑发展、资源高效利用。推进农村土地整理和生产经营组织形式创新，扩大集约化生产规模，叫响绿色产品品牌。大力推广“双向城市化”等五种城镇化建设模式，优先发展“一核三带”中心镇、节点镇。重点推进辽河区孤家子全国综合改革发展试点镇和铁东区叶赫省级示范城镇建设，着力打造少数民族特色村寨，加快确定10个市级城镇化示范镇，加大政策、资金等要素倾斜，全面落实放权强镇政策。做实做好城乡一体化公共服务。积极落实国家和省有关城乡改革政策和举措，让进城农民享有城镇居民同等的教育、住房、社保、医疗、计生等基本公共服务。下力气推进城乡基础设施共建共享，完善农村电、气、路、通信、饮水管网等生活配套设施，加快和完善城乡路网工程建设，有条件的地方稳步推进屯屯通、户户通水泥路，不断提高农村生活便利性和舒适度。

　　(四)提升宜居城市建设质量，打造城乡优良生态环境。继续推进“山水林路网”规划建设，实施蓝天工程、碧水工程、低碳工程、绿化工程、利民工程，提升城市质量和服务功能。全面启动PM2.5监测试点，加大机动车尾气污染检测防治力度，力争机动车环检达80%以上。严控城市建筑扬尘污染，开展加油站油气回收治理，推进小锅炉撤并改造，积极推进秸秆还田、气化等转化利用。尽快完成污水处理厂提标升级改造，抓紧建设南河、北河污水处理厂，让“两河”水活起来。完善大南山开发建设规划、北山绿化美化规划，易地规划建设殡仪馆，推进烈士陵园改造工程，做好凯旋公园建设前期准备。以“一湖两区”串连生态发展，打造“两河四岸五湖”等生态景观，加快建设四平西湖、南河、北河3处湿地，完成百公顷水面湿地建设任务。发挥绿化基金作用，完成各类造林15万亩，城区绿树进城30万株。搞好农村村旁、路旁、水旁、宅旁“四旁”绿化，改善农村生活环境。规划建设快速路简易立交桥、地上地下停车场、水冲厕所等公共服务设施，打通断头路、修复破损路，加快供热、燃气、供水、排水等市政管网维修改造步伐，做好零标高以下良心工程。启动城市视频数据共享交换管理平台、市民一卡通、智慧医疗、智慧产业等项目建设，加快智慧城市建设步伐。挖掘开发历史人文资源，建设叶赫满族风情园区、叶赫满族生态博物馆和叶赫生态影视城。突出抓好农村人居环境建设，继续开展“美丽乡村”评选活动，力争全市有60个村达到“美丽乡村”标准，每个县(市)区有1-2个集中连线的新农村精品样板村群。

　　(五)提升保障惠及民生实效，让百姓共享改革发展成果。按照守住底线、突出重点、完善制度、引导舆论的思路，全面推进“十五大民生工程”，继续扎实办好商、居、食、行、乐及教育、医疗、增收、养老、和谐等10个方面35件民生实事，预计投资总额30亿元。完善社区网格化管理，合理布局商业网点，形成城区便民千米生活圈，推进商业品质、服务提升。新建廉租住房750套、3万平方米，改造“暖房子”120万平方米，新建改造供热管网95公里，新增供热能力200万平方米。加强食品药品安全监管，开展诚信体系、可追溯体系建设，完善食品安全快速检测体系，扩大屠宰厂远程监控覆盖面，严厉打击违法违规行为。坚持城市公交优先战略，优化主城区公交布局，合理增加公交线路，实施过街天桥、地下廊道工程，实现四梨城际公交化。继续开展“文化七进”、“三下乡”等活动，丰富百姓精神文化生活。加快建设图书馆、博物馆、群众艺术馆和朝鲜族艺术馆，积极推进体育馆建设。谋划打造历史文化街区，启动恢复辽北大学，规划建设东山公园、“莱茵河畔”等工程。实施教育普惠工程，加快农村义务教育薄弱学校建设，促进义务教育均衡发展。拓展市区标准化幼儿园数量，大力发展农村学前教育，实现幼儿教育城乡全覆盖。进一步整合职业教育资源，加快组建职业教育集团。继续深化“四师一满意”主题教育活动，推进教育信息化建设步伐。积极做好医疗和生育保险工作，健全全民医保体系，全面实现省内异地就医异地即时结算。深入开展“四名”创建活动，为市区60岁以上老年人及相关弱势群体免费体检复查。完善企业职工工资正常增长和支付保障机制，落实城乡低保和农村五保供养标准自然增长机制，扶持大学生、农村转移劳动力、城镇困难人员、退役军人等重点人群的就业创业，鼓励城乡居民多渠道增加财产性收入。加快新农保和城居保制度合并实施，提高失业保险金发放标准，完善和落实被征地农民社会保障政策。推动市社会福利院改造工程，全面完成20所养老院改扩建任务，新建10个社区老年人日间照料中心。研究解决两区环卫临时工工资偏低问题，为特殊困难残疾人开辟公益性岗位，加大农村残疾人扶贫开发力度，解决就业和生活困难问题。

　　(六)提升社会治理运行水平，促进区域社会和谐稳定。切实创新社会治理方式，激发社会组织活力，实现由政府管理向政府、企业事单位、社会组织、基层自治组织等多元治理转变。统筹经济与社会、城市与乡村、政府与市场、条条与块块，实现全面协调可持续发展。加快政务信息化进程，培植信息产业发展，强化保障信息安全，全面提高社会事业信息化水平，为城市转型提供可靠支撑。着手制定项目建设“一费制”实施意见，积极防控债务风险，把财政专项资金、政府性投资项目集中监管工作纳入绩效考核范围。探索集中文明和谐执法，大力整治非法营运三轮车、路边烧烤、焚烧祭祀品以及侵占小区绿地行为，规范城市秩序，提高管理水平。以“安居乐业美好家园”为主题推进十星级创建，继续完善“五位一体”大调解机制。改革信访工作制度，完善领导干部接访下访机制，及时就地解决群众合理诉求。做好法律援助，以法治方式解决社会矛盾。进一步做好双拥工作，努力争创全国双拥模范城七连冠。完善社会治安防控体系和能力建设，严厉打击各类违法犯罪活动，坚决遏制严重刑事案件、重大群体事件，扩大“流动警务室”巡逻防控面，提升人民群众的安全感满意度。严格落实已经建立起来的安全生产长效机制，形成属地监管、行业监管、综合监管“三位一体”的监管格局，不间断地开展食品药品、农产品、公共卫生、道路交通、生产安全为重点的安全生产监管，严格落实企业主体责任，彻查隐患，不留死角。特别对老旧燃气管网和占压管网重点排查，未改造的53公里燃气管线全部改造完毕。防止发生重特大事故，确保生产安全、人民平安。

　　三、加强政府自身建设

　　面对新形势新任务，政府全体工作人员必须坚持为民、务实、清廉，切实做到对人民负责、受人民监督、让人民满意。

　　强化依法行政。自觉接受人大法律监督和政协民主监督，虚心接受舆论监督和群众监督。认真办理人大代表建议和政协委员提案。认真执行政府工作规则，健全科学依法民主决策机制，严格绩效考核，确保权责一致。积极推行公示、咨询、听证等制度，积极回应社会关切。推进依法行政考核，建立健全法律顾问制度，完善规范性文件、重大决策合法性审查机制，加强行政执法监督，完善行政复议机制，开展行政执法专题培训，做到严格规范公正文明执法。

　　转变政府职能。进一步简政放权，制定清理规范行政审批事项的实施意见，做到非行政许可审批项目“零审批”。加强政府公共服务、市场监管、社会管理、环境保护等职责。凡属事务性管理服务，原则上都要引入竞争机制，加大政府购买公共服务力度。继续抓好政府机构改革，稳步推进事业单位分类改革，积极探索有条件的事业单位转为企业或社会组织。完善政府信息公开、新闻发布、网络新闻发言人以及局长访谈制度，提升行政权力运行公开化水平。

　　密切联系群众。深入开展党的群众路线教育实践活动，大力整治“五风一顽症”，继续推行“一线工作法”，狠抓工作落实。大兴调查研究之风，深入基层群众，注重解决实际问题。强力推进“问政于民、职责公开”工作，推动网络问政和网上建言献策，切实做到直接面对群众、普遍联系群众、热心服务群众。凡是涉及群众利益的决策都要充分听取群众意见，凡是损害群众利益的做法都要坚决防止和纠正。

　　做到廉洁自律。坚持清廉从政、从严治政，加强政府机关廉洁从政教育，强化行政监察和审计监督。继续落实“5+1”制度，全面推进“五权”工作，加大对权力的制约和监督力度。严格执行党风廉政建设责任制和领导干部重大事项报告制度，加强领导干部经济责任审计，推进廉政风险防控。严格执行《党政机关厉行节约反对浪费条例》，进一步压缩行政开支，做到厉行节约、勤俭办事。

　　各位代表，党的十八届三中全会吹响了全面深化改革的号角。让我们在市委的坚强领导下，紧紧依靠全市人民，解放思想，改革创新，求真务实，奋力开拓，为早日实现四平赶超隆起、全面振兴而不懈奋斗!
